# Furry, Fomite, and Facultative Anaerobe: A Unique Case of Capnocytophaga canimorsus

**DOI:** 10.7759/cureus.47747

**Published:** 2023-10-26

**Authors:** Christopher J Peterson, William F Abel, Varsha Reddy, Kyle Pfaff

**Affiliations:** 1 Internal Medicine, Virginia Tech Carilion School of Medicine, Roanoke, USA

**Keywords:** sinusitis, immunocompetent, risk factors, canine (domestic), bacteremia, capnocytophaga canimorsus

## Abstract

*Capnocytophaga canimorsus* infection is frequently associated with dog and cat bites or scratches in patients who have risk factors such as immunosuppression, asplenia, and alcohol abuse. However, rare instances of *C. canimorsus* infection in patients without typical risk factors have been reported. Here, we present such a rare and unusual case of *C. canimorsus* bacteremia in a patient without animal wounds or risk factors. Chronic sinusitis may have contributed to mucosal disruption and served as an entry point for *C. canimorsus*. Prompt initiation of antibiotics resulted in rapid resolution of symptoms and clearance of bacteremia.

## Introduction

*Capnocytophaga canimorsus* is a gram-negative *Flavobacterium* that is generally found in the mouths of dogs and cats. It is a zoonotic pathogen that has been known to cause bacteremia, meningitis, endocarditis, and gangrene. It has mostly affected asplenic patients or those with a history of alcohol abuse (although infections in patients without these risk factors have also been documented). *C. canimorsus* is usually grown on media that is rich in iron such as sheep blood agar or chocolate agar, though its slow growth means microbiological diagnosis may take days and even weeks. Polymerase chain reaction is considered the gold standard method of identification. In the literature, transmission of the bacterium to humans has mostly been described as secondary to dog bites, scratches, or salivary contamination of wounds. Inoculation of the bacterium into the blood through means other than skin breakdown, as occurs in our case, is less common [1,2].

## Case presentation

An 86-year-old male with a past medical history significant for atrial fibrillation, gout, and multiple previous sinus infections and prior treatments (including remote turbinate surgery and polypectomy several years prior) presented with four days of malaise and fevers as high as 103°F. Initial examination revealed the patient to be alert and oriented, tachycardic, and febrile to 101.1°F, with minor tenderness over the maxillary sinus and no other significant findings. Admission labs revealed elevated inflammatory markers and a high-normal leukocyte count (Table 1).

**Table 1:** Admission labs CRP = C-reactive protein; ESR = erythrocyte sedimentation rate; AST = aspartate aminotransferase; ALT = alanine transaminase; PCR = polymerase chain reaction. Abnormal values are bolded.

Lab	Value	Reference range
Hemoglobin	12.3 g/dL	13.0 – 16.0 g/dL
White blood cell count	10.5 K/uL	4.0 – 10.5 K/uL
Platelet count	158 K/uL	130 – 500 K/uL
Creatinine	0.78 mg/dL	0.5 – 1.4 mg/dL
Sodium	132 mmol/L	135 – 145 mmol/L
Potassium	3.8 mmol/L	3.5 – 5.3 mmol/L
Glucose	93 mg/dL	70 – 99 mg/dL
Blood urea nitrogen	26 mg/dL	6 – 20 mg/dL
Total bilirubin	1.3 mg/dL	<1.3 mg/dL
AST	39 IU/L	10 – 42 IU/L
ALT	23 IU/L	10 – 60 IU/L
Alkaline phosphatase	101 IU/L	42 – 150 IU/L
Lipase	19 U/L	13 – 60 U/L
Lactic acid	1.9 mmol/L	1.9 mmol/L
CRP	32.48 mg/dL	<1.0 mg/dL
ESR	80 mm/HR	0 – 20 mm/HR
Influenza (PCR)	Not detected	Not detected
COVID-19 (PCR)	Not detected	Not detected

**Figure 1:**
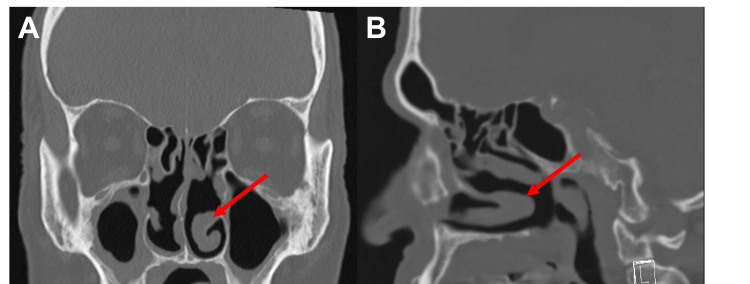
CT sinus revealing chronic sinusitis Chronic sinusitis and postsurgical changes with no findings to indicate superimposed acute sinusitis. Mucosal thickening bilaterally in the frontoethmoidal recesses is noted.

Though the patient lived at home with his pet dog, history and physical exam revealed no obvious bites, wounds, or scratches. He denied any dental issues. He denied any smoking history and endorsed two to three alcoholic drinks per week. He was not taking chronic steroids for gout, though he had a recent gout flare about 1.5 months prior treated with a five-day course of oral prednisone. CT of the abdomen and pelvis revealed no signs of intra-abdominal infection. CT sinus revealed postsurgical changes and chronic sinusitis (Figure 1) but no evidence of abscess or acute sinusitis. Though the initial concern was for sepsis, his clinical picture was more consistent with bacterial sinusitis, and he was started on empiric antibiotics (ampicillin-sulbactam and doxycycline).

The patient rapidly improved with antibiotic therapy and was discharged two days later with four days of amoxicillin-clavulanate and an outpatient referral for otolaryngology. However, the evening after discharge blood cultures grew gram-negative rods. Antibiotics were changed to a seven-day course of empiric levofloxacin and instructions to return to the emergency department should the patient develop fevers. Further growth identified *Capnocytophaga spp. *(4/4 bottles; sensitivities in Table 2) and, after discussion with an infectious disease consultant, outpatient antibiotics were again transitioned to amoxicillin-clavulanate for an additional seven days of therapy. Matrix-assisted laser desorption/ionization coupled with time-of-flight mass spectrometry (MALDI-TOF MS) subsequently identified *C. canimorsus*. A second set of blood cultures, taken after initiation of antibiotic therapy, remained without growth. On follow-up in the ambulatory setting, he endorsed the complete resolution of all symptoms without any recurrent fevers. Further discussion with the patient revealed that his pet dog is energetic and in addition to licking hands, may lick an individual’s face as well.

**Table 2:** Antimicrobial sensitivities MIC = minimum inhibitory concentration.

Antibiotic	MIC (mcg/mL)	Interpretation
Ampicillin/sulbactam	0.25	Susceptible
Clindamycin	<=0.016	Susceptible
Meropenem	0.032	Susceptible
Metronidazole	8	Susceptible
Penicillin	0.125	Susceptible

## Discussion

Since first being described in 1976, *C. canimorsus* has become closely associated with animal bites, particularly dog bites, in part due to colonization of dog and cat saliva [3]. One study of 484 patients with *C. canimorsus* infections noted that 60% were associated with dog bites, with an additional 24% associated with other dog contact such as scratches and licking [4]. Indeed, animal contact is considered the main source of* C. canimorsus* infections. Studies have examined the prevalence of *C. canimorsus* in canine oral flora, ranging from 57.5% to 73% [5-7]. Other risk factors include male sex, asplenia, alcoholism, cirrhosis, and corticosteroid use [4,8]. Sepsis is the most common presentation in reported infections [4]. Other complications include endocarditis, meningitis, and disseminated intravascular coagulation with purpura fulminans [9,10].

First-line treatment is commonly β-lactam/β-lactamase inhibition combinations owing to the high prevalence of β-lactamase production among *Capnocytophaga* organisms [11,12]. However, Chesdachai et al. note that β-lactamase production was rare among cases of *C. canimorsus,* and no treatment failure was noted with cephalosporin use [12]. *C. canimorsus* wounds typically have minimal inflammation [4], possibly due to the absence of its lipopolysaccharide with toll-like receptor 4 (TLR-4) receptors [9]. Virulence factors such as gliding motility (facilitating tissue translocation into the bloodstream), catalase production, and resistance to complement contribute to *C. canimorsus *pathogenicity [9]. The mortality rate can be as high as 31% [10], with up to 70.3% receiving ICU care [9]. However, most of these studies are based on systematic literature reviews and thus may be biased toward more severe infections.

We suspect that *C. canimorsus* was introduced to the nasal cavities via contact with the patient’s dog, likely through licking the patient’s face. Chronic sinusitis likely resulted in mucus membrane disruption that served as a point of entry for the bacterium into the bloodstream. To our knowledge, there are no other reports of *C. canimorsus* infections associated with sinus or nasal passage infections that are not associated with a bite or wound. Of note, *Capnocytophaga* spp. has been associated with sinusitis and other maxillofacial infections [13]. For example, *Capnocytophaga* spp. was isolated in a patient with chronic maxillary sinusitis secondary to complications from a tooth extraction [14]. *C. sputigena, *found in human oral flora, caused severe orbital cellulitis and subperiosteal abscess in a 15-year-old patient [15]. In another case, a neutropenic patient developed *Capnocytophaga* bacteremia presumably secondary to a sinus infection [16]. *Capnocytophaga* spp. was also isolated from a cat with chronic sinusitis and rhinitis [17]. Other atypical cases include an iatrogenic *C. canimorsus* meningitis following myelogram, with a possible contribution from staff who were dog owners [8]. Atypical cases may occur in patients without risk factors or known wounds from dog bites [9], magnifying the need for clinical suspicion in these cases. In one such case, an otherwise healthy 63-year-old male developed septic shock with multiorgan failure due to *C. canimorsus*; he had only been touched and licked by his pet dog and was without bites or scratches [18]. As in this case, *C. canimorsus* was an unexpected pathogen in a patient without typical risk factors.

## Conclusions

Here, we report a unique presentation of *C. canimorsus *infection in an immunocompetent patient without typical risk factors such as animal bites or wounds, asplenia, overt immunosuppression, or heavy alcohol use, though immunosenescence and a brief but recent course of steroids may have contributed as well. This work adds to a limited body of literature demonstrating that, in rare instances, *C. canimorsus* infection can occur in otherwise healthy individuals whose exposure to dogs may be as simple as being licked. Clinicians should consider *C. canimorsus* infection in patients with bacteremia and close contact with dogs, even if wounds and typical risk factors for infection are absent.
